# Single crown restorations supported by 6‐mm implants in the resorbed posterior mandible: A five‐year prospective case series

**DOI:** 10.1111/cid.12825

**Published:** 2019-07-28

**Authors:** Felix L. Guljé, Gerry M. Raghoebar, Arjan Vissink, Henny J. A. Meijer

**Affiliations:** ^1^ Department of Oral and Maxillofacial Surgery University Medical Center Groningen, University of Groningen Groningen The Netherlands; ^2^ Center for Dental Implants De Mondhoek Apeldoorn The Netherlands; ^3^ Center for Dentistry and Oral Hygiene, Dental School, Department of Implant Dentistry, University Medical Center Groningen University of Groningen Groningen The Netherlands

**Keywords:** posterior mandible, prospective study, short dental implants, single restorations

## Abstract

**Purpose:**

To assess clinical performance of single restorations supported by 6‐mm long implants in the posterior mandible after 5 years in function.

**Materials and Methods:**

Twenty‐one consecutive patients with the absence of premolars or molars in the posterior mandible and an estimated bone volume of at least 6 mm in width and an estimated height of 8 mm between the top of the ridge and alveolar nerve were included. Each patient received one or more 6‐mm implants. Custom‐made titanium abutments with cemented zirconia‐based porcelain crowns were placed after a 3‐month osseointegration period. Data of clinical examinations and radiographs were assessed at placement of the restoration and 12 and 60 months thereafter. The patients answered a questionnaire to score the satisfaction before treatment and after 12 and 60 months with the restoration in function.

**Results:**

Implant survival was 100%. Five‐years' mean marginal bone loss was 0.14 mm (SD: 0.4). Indices scores for plaque, calculus, gingiva, and bleeding were low as well as mean pocket‐probing depth. Patients' satisfaction was high.

**Conclusion:**

Five‐year follow‐up data of this limited case series study revealed that 6‐mm dental implants inserted in the resorbed posterior mandible provide a solid basis for single tooth restoration.

## 1 INTRODUCTION

In the posterior region of the mandible, the bone height above the mandibular nerve often frustrates the use of standard length implants (≥10 mm). Either surgical reconstruction of the planned implant site by vertical bone grafting techniques in combination with implants of standard length has to be applied or shorter implants should be used. Felice and colleagues^1^ and Esposito and colleagues^2^ stated being in favor of the use of shorter implants as such an approach reduces surgical interventions, treatment time and morbidity. Moreover, as it is a less complicated approach, the treatment outcome is presumed to be even more reliable.

In the systematic review of De N. Dias and colleagues,^3^ it was reported that survival rates of implants of ≤8 mm in length are comparable to those of longer implants in combination with vertical reconstructive surgery. Even more importantly, the use of short implants is presumed to be a significant asset in cases where there is a lack of bone for placement of longer implants in the posterior mandible as vertical bone augmentation procedures in that area should be avoided.^2^ Another recent review even states that in case of limited mandibular bone height short implants are favored because of a number of advantages for the patients and the clinician.^4^ Prospective studies with a medium‐term and long‐term follow‐up on short implants of ≤8 mm in the posterior mandible are scarce, however.

Clinical studies with 5‐year results on performance of short implants in the resorbed posterior mandible are limited to those of Rossi and colleagues,^5^, ^6^ Pieri and colleagues^7^ and Naeni and colleagues.^8^ All these studies but one reported the results of a mixture of treatments with short implants in mandible and maxilla. Overall survival rates varied from 86.7% to 95.0%. Due to a difference in bone density, it is not yet shown whether the performance of short implants differs between maxilla and mandible. Only the retrospective study of Pieri and colleagues^7^ solely reported on implant treatment in the posterior mandible. The implant‐survival rate in that 5‐years study was 97.8%. Rossi and colleagues^9^ are the only authors reporting about 10‐year results on short implants in the posterior maxillary and mandibular region. The 10‐year overall survival rate of implants placed in either the maxilla or mandible was 91.7%. A prospective medium‐term study, solely focusing on short implants in the resorbed posterior mandible, is missing. Therefore, the present case series study was performed to evaluate the clinical performance after 5‐years in function of 6 mm implants restored with non splinted crowns in the posterior region in the mandible. The primary objective of the study was marginal bone level changes by radiological assessments at 5‐year follow‐up. Secondary objectives were patients' satisfaction, implant and restoration survival and condition of peri‐implant mucosa.

## 2 MATERIALS AND METHODS

The treatment and evaluation procedures applied in this study have been described in detail in the 1‐year study of Guljé and colleagues.^10^ A summary of the procedures utilized is presented below.

### 2.1 Inclusion criteria

During a 2‐year inclusion period, consecutive patients, with one or more missing teeth in the (pre)molar region of the mandible with a bone width of at least 6 mm and a bone height above the mandibular nerve of 8 mm were selected to participate in the study if the inclusion and exclusion criteria met. The screening procedure included a clinical and radiographic examination (intraoral radiographs and dental panoramic).

When meeting the inclusion criteria and none of the exclusion criteria patients were included in the study after signing the informed consent form. The design was a two‐center case series study (University Medical Center Groningen and private practice “De Mondhoek” Apeldoorn). The Medical Ethical Committee of the University Medical Center Groningen, considered this case series study was considered not to be subject to the Medical Research Involving Human Subjects Act (Number M13.139273).

### 2.2 Surgical and prosthetic procedures

**Figure 1:**
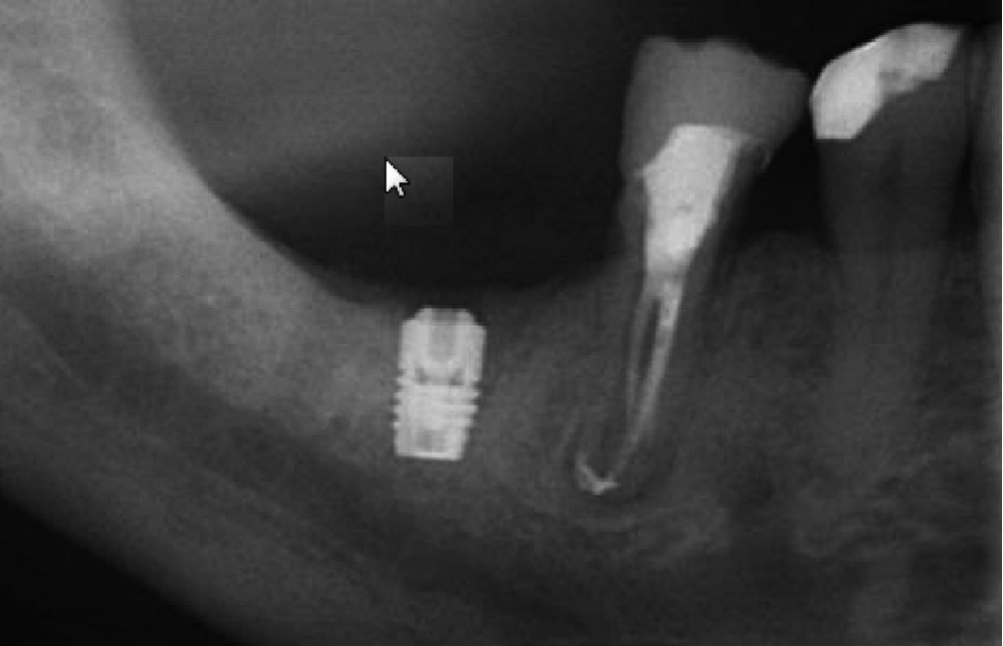
Part of rotational panoramic radiograph with 6‐mm implant in position 46, 2 weeks after implant placement

Implant surgery was performed using the standard Astra Tech Implant System protocol (document 79 254‐usx‐1002 Astra Tech 2010). The surgical procedure was performed under local anesthesia in Apeldoorn by F.L.G. and in Groningen by G.M.R. After a crestal incision, buccal and lingual flaps were raised. A 6‐mm implant (OsseoSpeed 4.0 S, Astra Tech Implant System, Dentsply Implants, Mölndal, Sweden) was placed. The implants were placed submucosal (Figure 1). After a 12‐week healing period, the second stage surgery was performed and a healing abutment was placed. In Apeldoorn, implant surgery was performed by F.L.G. and in Groningen by G.M.R.

Two weeks after the second‐stage surgery an impression at implant level was made in order to manufacture the crown and abutment restorations. Placement of the titanium individual abutment (Atlantis abutment, Dentsply Implants) at 20 Ncm torque with cementation of the zirconia‐based porcelain crown was 2 weeks after the impression was made.

### 2.3 Outcome measures

Throughout the 5‐year follow‐up period the following outcome measures were assessed at the evaluation time points (preoperatively, 2 weeks (T1), 12 months (T12), and 60 months (T60) after restoration placement):

- Implant survival: implant still present, not mobile and removal not dictated by progressive bone loss, infection or fracture.
- Restoration survival: restoration still present, not renewed and renewal not dictated by extensive fracture or inferior aesthetics.
- Radiographic evaluation: radiographs were taken with an individualized X‐ray holder to make the radiographs individually comparable. Crestal bone changes were measured, both distally and mesially, from a reference point to the crestal bone margin. The reference point was the junction between the machined bevel and the micro threads at the neck of the implant. Bone loss was presented as the worst value for mesial and/or distal changes between 2 weeks, 12 months, and 60 months after restoration placement.
- Clinical evaluations: plaque accumulation was measured with the modified Plaque Index^11^ and bleeding tendency with the modified Sulcus Index,^11^ assessment of peri‐implant inflammation according the Gingival Index,^12^ presence of dental calculus and pocket probing depth to the nearest millimeter using a manual periodontal probe.
- crown‐implant ratio: clinical crown‐implant ratios were calculated on digitized casts as described by Meijer et al.^13^ and Guljé et al.^14^
- Patient satisfaction: patients validated the treatment result with an overall mark (on a 10‐point rating scale) and were asked to answer a questionnaire composed of questions or statements on a 5‐point rating scale ranging from (score 1) “very satisfied” and “in agreement” to (score 5) “very disappointed” and “not in agreement”.

### 2.4 Statistical method

The same observer (F.L.G.) did analysis of the radiographs and data collection. The worst score per implant of the radiographic and clinical parameters were used in the data analysis and presented as frequencies. Differences in peri‐implant bone changes and pocket probing depth between time periods were tested with the Paired Samples *t*‐test. Differences in patients' satisfaction between pretreatment, 1‐year and 5‐year follow‐up were tested with the Wilcoxon signed‐rank test. Analysis was done with the Statistical Package for Social Sciences (version 23.0 SPSS Inc., an IBM Company, IBM Corporation, Chicago, Illinois). In all tests, a significance level of 0.05 was chosen.

## 3 RESULTS

All eligible consecutive patients agreed to participate in this study. A total of 21 patients (7 males and 14 females, mean age 57.3 years, range 44‐70 years) were included. During the 2‐year inclusion period, most patients could not be included because bone height above the mandibular nerve exceeded 8 mm. These patients received longer implants. The included 21 patients received 31 implants. Patient characteristics are depicted in Table 1. All patients completed the 5‐year evaluation period and joined the last follow‐up visit.

**Table 1:** Baseline characteristics of study group with 21 patients and 31 implants

Mean age in years (SD, range)	57 (9.1, 44‐70)
Gender (number male/female)	7/14
Implant position (number premolar/M)	12/19
Implant position (between teeth/no tooth distally)	21/10

**Figure 2:**
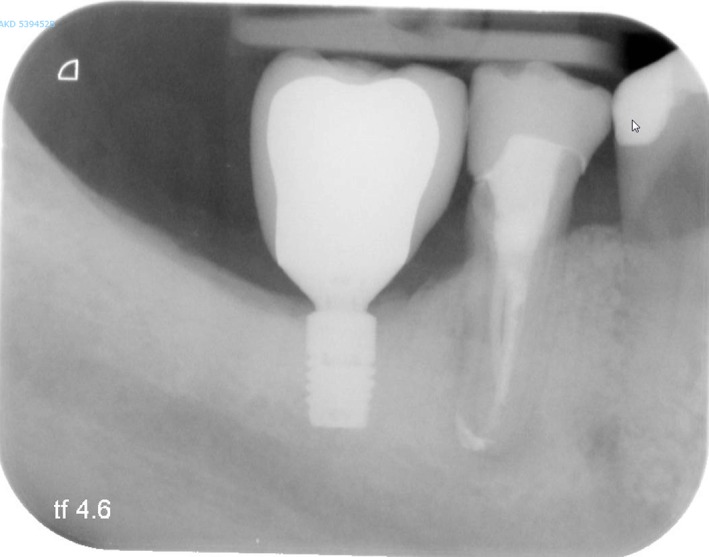
Intraoral radiograph of 6‐mm implant with restoration, 1 year after restoration placement (same patient as depicted in Figure 1)

**Figure 3:**
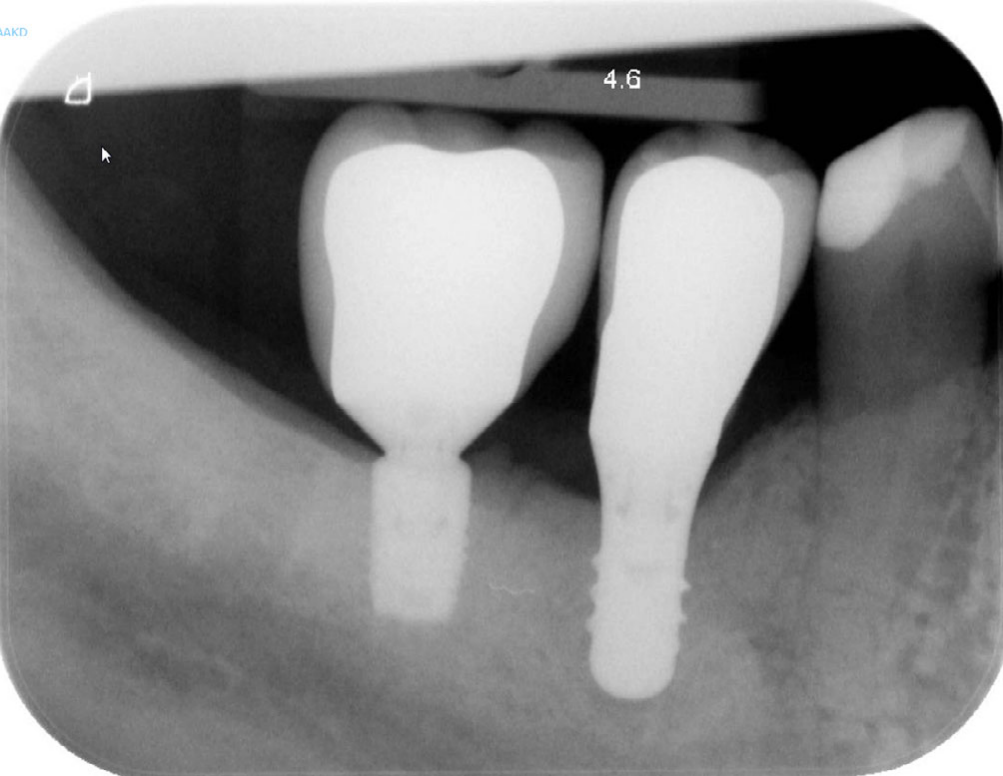
Intraoral radiograph of 6‐mm implant with restoration, 5 years after restoration placement (same patient as depicted in Figure 1)

No loss of implants or restorations had occurred during the 60‐month follow‐up. The mean loss of marginal bone at T60 was 0.14 ± 0.38 mm; on average no additional loss of marginal bone was observed at the 5‐years follow‐up (Table 2) (Figures 2 and 3). Scores of the indices for plaque, calculus, gingiva, and bleeding were low and did not change over time (Table 3). Also, mean probing depth was favorable (2.6 ± 0.7 mm) and did not change during follow‐up. Mean crown‐implant ratio was 2.23 with a SD of 0.40. No technical complications (eg, porcelain chipping, screw loosening) and no biological complications (eg, peri‐implantitis) were encountered during the 5‐year follow‐up. Patient's satisfaction was very high after treatment and remained at that high level during follow‐up (Table 4).

**Table 2:** Mean value and SD and frequency distribution (percentages) of marginal bone change (implant‐based) after 1 year (T12) and after 5 years (T60) in function

Bone change (mm)	T_12_ (n = 31)	T_60_ (n = 31)
Mean (SD)	−0.14 mm (0.42)	−0.14 mm (0.38)
> −2.0 up to and including −1.5	0 (0.0)	1 (3.2)
>−1.5 up to and including −1.0	3 (9.7)	0 (0.0)
>−1.0 up to and including −0.5	1 (3.2)	3 (9.7)
>−0.5 up to and including 0.0	25 (80.6)	25 (80.6)
>0.0 up to and including 0.5	1 (3.2)	2 (6.5)
>0.5 up to and including 1.0	1 (3.2)	0 (0.0)

No significant differences (Paired samples *t*‐test) between evaluation time periods (*P* = .978).

**Table 3:** Frequencies and percentages (implant‐based) of plaque‐index scores (possible score 0‐3), calculus‐index scores (possible score 0‐1), gingival‐index scores (possible score 0‐3), bleeding‐index scores (possible score 0‐3) and mean value, SD and minimum‐maximum value of probing depth (in mm) at 1 month (T1), 1 year (T12), and 5 years (T60) after placement of the restoration

	T_1_	T_12_	T_60_
Plaque‐index	Score 0: 31 (100%)	Score 0: 28 (90.3%) Score 1: 3 (9.7%)	Score 0: 27 (87.1%) Score 1: 4 (12.9%)
Calculus‐index	Score 0: 31 (100%)	Score 0: 31 (100%)	Score 0: 31 (100%)
Gingival‐index	Score 0: 30 (96.8%) Score 1: 1 (3.2%)	Score 0: 29 (93.5%) Score 1: 2 (6.5%)	Score 0: 29 (93.5%) Score 1: 2 (6.5%)
Bleeding‐index	Score 0: 23 (74.2%) Score 1: 7 (22.6%) Score 2: 1 (3.2%)	Score 0: 21 (67.8%) Score 1: 10 (32.2%)	Score 0: 21 (67.8%) Score 1: 9 (29.0%) Score 2: 1 (3.2%)
Probing depth in mm (sd), min‐max	2.4 (0.6), 1–4	2.5 (0.6), 2‐4	2.6 (0.7), 2‐5

*Note*: No significant differences (Paired samples *t*‐test) in probing depth between evaluation time points (T_1_‐T_12_: *P* = .374; T_1_‐T_60_: *P* = .147; T_12_‐T_60_: *P* = .403).

**Table 4:** Patient's satisfaction before treatment (Tpre), after 1 year (T12) and after 5 years (T60) and significant differences between time periods

	T_pre_ % in agreement	T_12_% in agreement	T_60_% in agreement
	(21 patients; 31 implants)	(21 patients; 31 implants)	(21 patients; 31 implants)
Feelings			
Presence of shame	19.4	0.0^a^	0.0^b^
Self‐confidence decreased	19.4	0.0^a^	0.0^b^
Visible being partial edentulous	25.8	0.0^a^	0.0^b^
Function			
Evade eating with the edentulous zone/implant	80.6	3.2^a^	6.5^b^
The ability to chew is decreased	83.9	3.2^a^	0.0^b^
Implant does influence the speech	‐	0.0	0.0
Implant does influence the taste	‐	0.0	0.0
Aesthetics			
Not satisfied with the color of the crown	‐	0.0	0.0
Not satisfied with the form of the crown	‐	0.0	0.0
Not satisfied with the color of the mucosa around the crown	‐	0.0	0.0
Not satisfied with the form of the mucosa around the crown	‐	0.0	0.0
Overall satisfaction (0‐10)	5.6 ± 1.5	9.3 ± 0.9^a^	9.6 ± 0.7^b^

a Significant differences T12 compared with pretreatment values (Wilcoxon signed‐rank test; *P* = 0.000‐0.003).

b Significant differences T60 compared with pretreatment values (Wilcoxon signed‐rank test; *P* = .000‐.002).

## 4 DISCUSSION

In the present study placement of 6‐mm implants in the posterior region of a resorbed mandible appears to be a solid solution to support single restorations. The implant survival rate after 5‐year was 100%, marginal bone loss was minimal, peri‐implant health favorable and patients' satisfaction high.

The high implant survival in the present study is the best comparable with results of the retrospective study of Pieri and colleagues^7^ that solely reported on implant treatment in the posterior mandible. The implant‐survival rate in that 5‐years study was 97.8%. However, in the latter study the short implants were splinted to neighboring implant‐supported restorations. The high density of mandibular bone, and therefore a high bone‐to‐implant contact area, could be a reason for the high survival rate in both studies. Also, restoration survival was 100%, which favorable outcome is, probably, due to the materials used and the firm connection between implant and abutment leading to reduction of major complications. Lemos and colleagues^15^ reported in their systematic review that restoration failures were most often associated with failure of the implants. Since in the present study no implants were lost, the high restoration survival rate is in line with the literature. Mean 5‐years loss of marginal bone was very low, being 0.14 mm. Felice and colleagues^16^ concluded in their 5‐year findings that short implants experienced statistically significantly less bone loss than longer implants. Other 5‐year studies with 6‐mm implants, in maxilla and mandible, reported bone loss varying from 0.18 to 0.7 mm.^5^, ^6^, ^7^, ^8^ In the present study and in the study of Pieri and colleagues^7^ bone level implants were used, whereas in the other 6‐mm studies tissue level implants were applied. In earlier days, a microgap at bone level was seen as a risk for bone loss, but this was refuted in the systematic review of Vouros and colleagues.^17^ Also in comparing peri‐implant bone changes of 6‐mm bone level implants with 6‐mm tissue level implants after 5 years, it seems that there are no relevant differences. Apparently, the close connection with platform switch of bone level implant and abutment, together with an optimum surface roughness at the neck of the implant, provides a stable peri‐implant marginal bone level. This is consistent with the favorable 5‐years results of platform switching in the study of Telleman and colleagues.^18^

Scores for plaque, calculus, gingiva, and bleeding were very low at the 5‐year evaluation. The strict oral hygiene regime to which patients were subjected provided healthy peri‐implant tissues. This favorable outcome also matches the low mean probing depth of 2.6 mm. Again these data support those of previous studies.^5^, ^6^

The rather high crown‐implant ratio of 2.23 (crown length more than twice as much as the length of the implant situated in bone) does not seem to have an impact on the presence of biological and technical complications after 5 years of function, since peri‐implant bone loss was very limited and technical complications were not absent.

Patients' satisfaction was high and remained at a high level during follow‐up. None of the other 6‐mm studies reported on patient satisfaction, making direct comparison impossible. However, also in the systematic review of Thoma and colleagues^19^ these high satisfaction scores with short implants were mentioned.

Vertical augmentation in the mandible can be accompanied by some complications such as failure of the augmentation procedure, infection, and nerve injury.^2^ Thus, when vertical augmentation surgery can be avoided, morbidity, risk, and costs will be reduced too. A limitation of the present study is that it was not designed as a randomized clinical trial with an augmentation procedure and placement of longer implants as a control group. However, reported complications and the rate of resorption of a vertical augmentation made a design with an augmentation group as a control ethically questionable to our opinion. Nevertheless, the medium‐term results of the present study support the use of short implants since it offers excellent results with a simple and safe treatment procedure as well as that no complications were observed.

Another limitation of this study is that a limited sample size was used. Although medium results are excellent, more studies with possibly larger patient populations are needed to strengthen the conclusions.

## 5 CONCLUSION

Within the limitations of this study, the 6‐mm OsseoSpeed 4.0 S implants with a single restoration placed in the posterior resorbed mandible provide a stable solution with healthy peri‐implant soft tissues and a high patient satisfaction after a 5‐years follow‐up period.

## CONFLICT OF INTEREST

This two‐center study has been partially sponsored by Dentsply Implants. None of the authors have economical interest in the product related in this study or in the company.
